# Genetic diversity in tef [*Eragrostis tef* (Zucc.) Trotter]

**DOI:** 10.3389/fpls.2015.00177

**Published:** 2015-03-26

**Authors:** Kebebew Assefa, Gina Cannarozzi, Dejene Girma, Rizqah Kamies, Solomon Chanyalew, Sonia Plaza-Wüthrich, Regula Blösch, Abiel Rindisbacher, Suhail Rafudeen, Zerihun Tadele

**Affiliations:** ^1^National Tef Research Program, Debre Zeit Agricultural Research Center, Ethiopian Institute of Agricultural ResearchDebre Zeit, Ethiopia; ^2^Crop Breeding and Genomics, Institute of Plant Sciences, Department of Biology, University of BernBern, Switzerland; ^3^National Agricultural Biotechnology Laboratory, Holetta Agricultural Research Center, Ethiopian Institute of Agricultural ResearchHoletta, Ethiopia; ^4^Plant Stress Laboratory, Department of Molecular and Cell Biology, University of Cape TownCape Town, South Africa

**Keywords:** *Eragrostis tef*, diversity, genomics, proteomics, tef, transcriptomics, variability

## Abstract

Tef [*Eragrostis tef* (Zucc.) Trotter] is a cereal crop resilient to adverse climatic and soil conditions, and possessing desirable storage properties. Although tef provides high quality food and grows under marginal conditions unsuitable for other cereals, it is considered to be an orphan crop because it has benefited little from genetic improvement. Hence, unlike other cereals such as maize and wheat, the productivity of tef is extremely low. In spite of the low productivity, tef is widely cultivated by over six million small-scale farmers in Ethiopia where it is annually grown on more than three million hectares of land, accounting for over 30% of the total cereal acreage. Tef, a tetraploid with 40 chromosomes (2n = 4x = 40), belongs to the family Poaceae and, together with finger millet (*Eleusine coracana* Gaerth.), to the subfamily Chloridoideae. It was originated and domesticated in Ethiopia. There are about 350 *Eragrostis* species of which *E. tef* is the only species cultivated for human consumption. At the present time, the gene bank in Ethiopia holds over five thousand tef accessions collected from geographical regions diverse in terms of climate and elevation. These germplasm accessions appear to have huge variability with regard to key agronomic and nutritional traits. In order to properly utilize the variability in developing new tef cultivars, various techniques have been implemented to catalog the extent and unravel the patterns of genetic diversity. In this review, we show some recent initiatives investigating the diversity of tef using genomics, transcriptomics and proteomics and discuss the prospect of these efforts in providing molecular resources that can aid modern tef breeding.

## Introduction

Tef [*Eragrostis tef* (Zucc.) Trotter] is the major food crop in Ethiopia where it is annually cultivated on more than three million hectares of land (CSA, 2014). Compared to other cereals, tef is more tolerant to extreme environmental conditions especially to water-logging. It is unique in its ability to grow and yield on poorly drained Vertisols which most cereals cannot tolerate. Unlike other cereals, the seeds of tef can be easily stored under local storage conditions without losing viability since the grains are resistant to attack by storage pests (Ketema, 1997). Tef grain is also a rich source of protein and nutrients and has additional health benefits including that the seeds are free from gluten (Spaenij-Dekking et al., 2005). According to a recent study, the bio-available iron content was significantly higher in tef bread than in wheat bread (Alaunyte et al., 2012). In general, tef provides quality food and grows under marginal conditions, many of which are poorly suited to other cereals. However, tef is considered to be an orphan crop since it is only of regional importance and has until recently not been the focus of crop improvement (Naylor et al., 2004; Assefa, 2014).

Despite its versatility in adapting to extreme environmental conditions, the productivity of tef is very low with the national average standing at 1.5 t/ha (CSA, 2014). Tef’s major yield limiting factors are lack of cultivars tolerant to lodging, drought, and pests (Assefa et al., 2011). Lodging is the permanent displacement of the stem from the upright position. Tef possesses tall, weak stems that easily succumb to lodging caused by wind or rain. In addition, lodging hinders the use of high input husbandry since the application of increased amounts of nitrogen fertilizer to boost the yield results in severe lodging. When this occurs, both the yield and the quality of the grain and the straw are severely reduced and both manual and mechanical harvesting are impeded. Various attempts have been made by the research community to develop lodging-resistant tef cultivars (Assefa et al., 2011; Tadele and Assefa, 2012) but presently no cultivar with reasonable lodging resistance has been obtained.

The analysis of genetic relationships amongst tef varieties is an important component of improvement programs because it provides information about the genetic diversity of the crop and sets a platform for stratified sampling of breeding populations. Tef represents a unique biodiversity component in the agriculture and food security of millions of farmers in Ethiopia. The conservation, characterization, and utilization of the existing tef genetic diversity are becoming increasingly important in view of the evolving needs and manifold challenges of small-scale farmers in Ethiopia. This is primarily because tef has remarkable genetic traits useful for most Ethiopian farmers to utilize for coping with erratic climatic conditions, generation of household income, and fulfilling concerns of nutritional needs. Moreover, the conservation and utilization of the tef genetic resources offer a reliable basis for enhancing food security and developing crop diversification in the moisture stress and challenging agro-ecological areas of the country.

Here, we present an overview of the results of the major studies made on tef diversity and recent initiatives underway to better understand the diversity at molecular level and utilize these diversities in improving the crop using modern genetic and genomic tools.

## Taxonomy and Accessions of Tef

Tef belongs to the Poaceae or Grass family as do all economically important cereals. It is closely related to finger millet (*Eleusine coracana* Gaerth.) as both are in the subfamily Chloridoideae. The genus *Eragrostis* comprises about 350 species from which only tef is cultivated for human consumption. Unlike wheat, barley and rice, which are all C_3_ plants, tef (along with maize and sorghum) is a C_4_ plant which efficiently utilizes carbon dioxide during photosynthesis. This can be seen by tef’s Kranz-type leaf anatomy with vascular centers surrounded by bundle sheath cells containing a high number of chloroplasts and by the low CO_2_ compensation point of the leaves, also typical of C_4_ as opposed to C_3_ species (Kebede et al., 1989).

Tef is an allotetraploid (2n = 4x = 40). Over the past few decades the ancestry of tef has been investigated using morphological and cytogenetic methods (Jones et al., 1978), biochemical methods (Bekele and Lester, 1981), and phylogenetic analysis using ribosomal DNA and transcription factor genes (Espelund et al., 2000) or nuclear and plastid genes (Ingram and Doyle, 2003). It has been suggested that *Eragrostis pilosa* is closely related to tef while *E. heteromera* and *E. cilianensis* are more distantly related (Ingram and Doyle, 2003). Similar conclusions were reached using biochemical methods (Bekele and Lester, 1981). The close relationship between tef and *E. pilosa* is also evidenced by the successful hybridization of these two species (Tefera et al., 2003a). This hybridization generated viable offspring and ultimately resulted in the release in 2009 of a variety called *Simada* (DZ-Cr-285 RIL295) from the inter-specific hybrid of tef [DZ-01-2785 × *E. pilosa* (line 30-5); MoA, 2013]. However, since *E. pilosa*, like tef, is a tetraploid, the diploid ancestors of tef remain unknown.

Ethiopia is the origin and center of diversity for tef (Vavilov, 1951), harboring landraces with a wide array of phenotypic diversity, and also wild progenitors and related wild species. Charring experiments suggest that the domestication history of tef might be different from that of barley and wheat since in some cases tef might not survive the high temperatures tolerated by other cereals (D’Andrea, 2008).

As in any crop improvement program, tef breeding also relies mainly upon the germplasm resources existing in the genetic stock. Diverse types of accessions are available in the country, and collection, evaluation, and utilization of tef germplasm by national and international groups began in Ethiopia in the late 1950s. However, organized collection at the national level was made after the establishment of the Plant Genetic Resources Center of Ethiopia (PGRC/E) in 1976. After several changes in its name and mandate, the institute responsible for germplasm collection and maintenance as well as distribution is currently called the Ethiopian Institute of Biodiversity (EIB). The institute with only 1067 tef accessions in Demissie (1991) has reached to 5169 accessions in 2011 (Tesema, 2013). This fourfold increase in the collection size in just two decades shows the presence of both a wide diversity of germplasm in the country and also the commitment of institutes and individuals to collect and preserve these germplasm for future use.

Characterization of the accessions according to their properties such as morphology is important in order to provide information to interested researchers or other sectors of society. The first and most comprehensive detailed morphological descriptions for 35 tef cultivars were given based on phenology, plant vigor, shoot and root related traits, panicle form, spikelet size, growth habit, and lemma and caryopsis color (Ebba, 1975; **Table 1**).

**Table 1:** Selected properties of 35 tef ecotypes (cultivars) characterized (Ebba, 1975).

No	Ecotype (cultivar) name	Seed color	Plant height (cm)	Panicle		Days to		No. of spikelets per panicle	No. of florets per spikelet
				Length (cm)	Form	Heading	Maturity		
1	Ada	yWh	80	31	S-comp	45–50	95–115	320	8.3
2	Addisie	yWh	80	30	V-comp	45–50	95–110	310	6.0
3	Adoensis	mBr	70	30	V-loose	45–50	90–95	440	6.5
4	Alba	yWh	85	45	F-loose	45–50	95–120	525	10.0
5	Balami	yWh	88	36	V-loose	40–45	90–110	424	8.0
6	Beten	yWh	70	30	V-loose	40–45	85–95	220	7.0
7	Bunninye	mBr	34	16	V-loose	35–40	75–85	90	6.0
8	Burssa	yWh	58	20	S-comp	45–50	85–90	210	7.4
9	Curati	poW	88	40	S-comp	50–60	95–120	600	6.5
10	Dabbi	mBr	70	30	V-loose	40–45	80–95	295	6.5
11	Denekye	mBr	60	20	S-comp	45–50	90–115	165	9.6
12	Dschanger	mBr	75	30	F-loose	40–45	90–110	210	6.2
13	Enatitie	yWh	70	30	V-loose	40–45	90–100	270	6.2
14	Fesho	Br	50	20	V-loose	38–45	75–85	135	6.2
15	Gea-Lamie	Br	30	15	V-loose	25–30	60–70	60	6.8
16	Gofarie	yWh	78	28	S-comp	45–50	90–100	285	7.0
17	Gommadie	yWh	75	25	S-comp	45–50	90–100	290	8.9
18	Gorradie	yWh	90	40	V-comp	50–55	95–120	356	6.8
19	Hamrawe Murri	yWh	75	30	V-comp	50–55	90–100	310	6.9
20	Hatalla	yWh	90	38	V-loose	50–55	90–115	420	6.7
21	Janno	yWh	75	30	F-loose	45–50	85–105	335	8.5
22	Karadebi	Br	55	22	V-loose	40–45	85–90	160	6.7
23	Kaye Agachem	lBr	77	30	V-comp	45–50	90–110	300	6.8
24	Kaye Murri	yWh	80	30	V-comp	45–50	90–105	280	6.5
25	Manya	yWh	75	35	F-loose	40–45	90–110	350	9.0
26	Murri	yWh	95	38	V-comp	50–55	105–120	330	5.0
27	Purpurea	Br	85	38	F-loose	45–50	90–105	285	7.7
28	Rosea	yWh	75	30	F-loose	45–50	90–100	215	10.7
29	Rubicunda	yWh	85	35	F-loose	45–50	90–115	290	8.4
30	Shawa Gemerra	Br	35	16	F-loose	30–35	60–75	60	12
31	Trotteriana	Br	70	25	V-comp	50–55	90–95	210	8.0
32	Tullu Nasy	poW	42	17	V-loose	35–40	60–70	115	6.3
33	Variegata	lBr	70	32	F-loose	45–50	90–100	160	10.9
34	Viridis	poW	75	35	F-loose	45–50	85–95	275	6.7
35	Zuccagniana	Br	65	27	V-comp	45–50	90–100	200	6.4

*Panicle form*: V-loose, very loose; F-loose, fairly loose; S-comp, semi-compact; V-comp, very compact. *Seed color*: Br, brown; mBr, medium brown; lBr, light brown; poW, purple orange white; yWh, yellow white.

## Phenotypic Diversity in Tef

Tef is highly diverse and variable in terms of morphological and agronomic characters. The distribution of the crop in different agro-ecological zones coupled with the selection by farmers on the basis of their preferred traits has resulted in a number of varieties with unique characters. Genetic diversity analysis of tef accessions facilitates the development of improved varieties with high productivity and yield stability. In view of this fact, efforts have been made to assess and quantify the extent and pattern of genetic diversity in the tef germplasm collections using different approaches (**Table 2**).

**Table 2:** Studies made on phenotypical and morphological diversity in tef.

Germplasm or genotypes		Sites (No)	Reference
Type	Number		
Natural accession	124	1	Mengesha et al. (1965)
Hybrids	559	1	Berhe et al. (1989a,b,c)
Natural accession	21		Bekele (1996)
Natural accession	225		Ketema (1993)
RIL (key Murri × Fesho)	165	3	Tefera et al. (2003b)
RIL (key Murri ×*E. pilosa*)	200	3	Tefera et al. (2003a)
F_2_ (12 crosses)	12	1	Tefera et al. (2008)
Natural accession	320	2	Assefa et al. (1999, 2000)
Natural accession	120	4	Assefa et al. (2001b)
Natural accession	1080	1	Assefa et al. (2001a)
Natural accession	3000	1	Assefa et al. (2002a,b, 2003b)
Natural accession	3600	1	Kefyalew et al. (2000)
Natural accession	144	2	Adnew et al. (2005)
RIL (196 × 974)	196	2	Chanyalew et al. (2006)
RIL (196 × 2356)	190	2	Chanyalew et al. (2009)
RIL (Kay Murri ×*E. pilosa*)	94	3	Yu et al. (2007)
Natural accession	37	1	Ayalew et al. (2011)
Natural accession	15	1	Plaza-Wüthrich et al. (2013)
Natural accession	81	1	Lule and Mengistu (2014)

RIL: recombinant inbred lines.

### Diversity in Natural Populations

**FIGURE 1:**
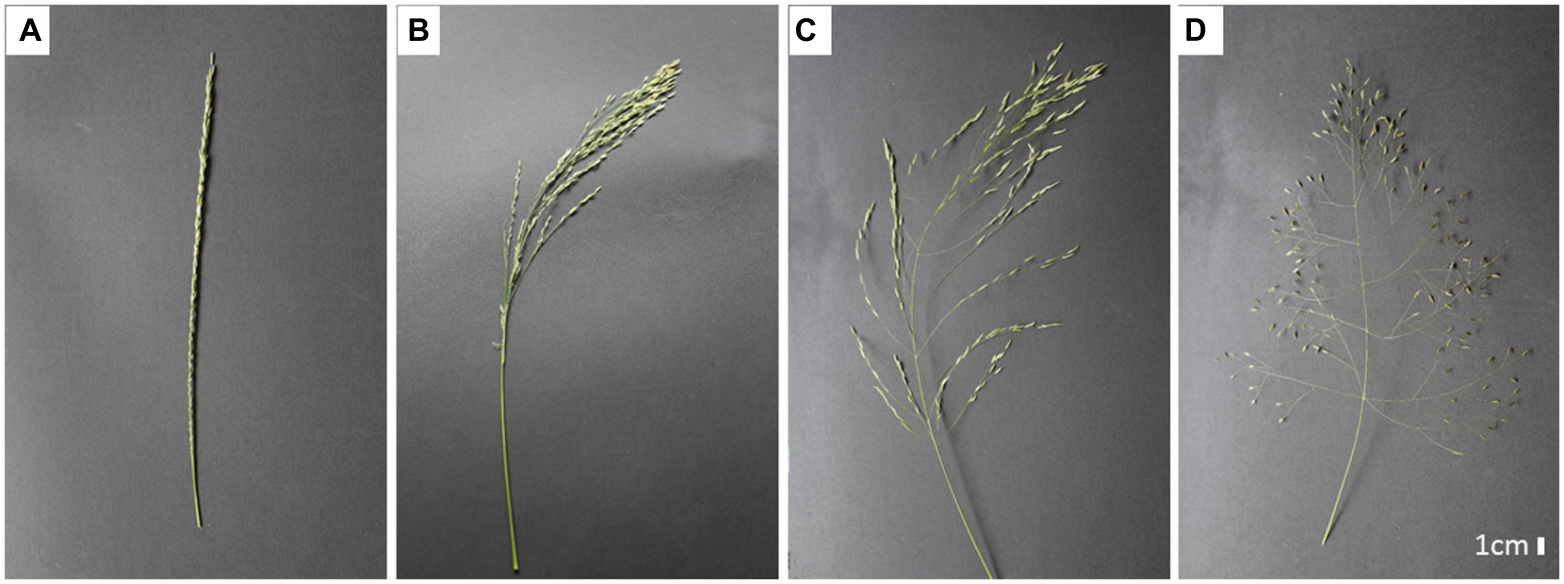
**Diversity in the form of tef panicles. (A)** Very compact, **(B)** semi-compact, **(C)** fairly loose, **(D)** very loose.

The first studies on phenotypic diversity in tef germplasm used 124 single panicles collected from the major tef producing areas in Ethiopia as a source of seed. The germplasm accessions showed significant variability for plant height, panicle length, maturity, seed color, seed yield, lodging, and panicle form (Mengesha et al., 1965). As shown in **Figure 1**, at least four distinct panicle forms are present in tef accessions, namely very-compact, semi-compact, fairly loose, and very-loose.

Later, studies involving 2255 tef lines collected from different parts of the country showed high variation for flag leaf area, single plant grain yield, and straw yield (Ketema, 1993). The analyses of 9885 accessions collected from 14 former provinces of Ethiopia showed simple coefficient of variation (SCV) estimates ranging from 32% for primary panicle branches to 217% for grain yield/plant (Bekele, 1996). While using SCV, the extent of variation among traits is not affected by the magnitudes of values and units of measurement. Since SCV does not efficiently measure diversity among traits, phenotypic (PCV) and genotypic (GCV) coefficients of variation, which are based on partitioning of the total variance into components of genetic and non-genetic factors, are now more extensively used. Accordingly, various breeders have applied these two indices in evaluating the tef germplasm (Tefera et al., 1990; Hundera et al., 1999; Assefa et al., 2000; Chanyalew et al., 2009). Most of these studies revealed significant to highly significant differences among the genotypes for most of the traits examined, and this variability would serve as a basis for the improvement of the crop. Because the magnitude of genetic variation is better assessed from GCV, breeders usually focus on traits with high GCV estimates. High GCV values were reported for tiller number, panicle weight, grain yield per panicle, plant biomass, and grain yield (Assefa et al., 1999, 2001b; Balcha et al., 2003; Tefera et al., 2008; Chanyalew et al., 2009). This wide genetic variation indicates much potential for improving the crop through direct selection and/or hybridization.

Characters with huge variability include: days to panicle emergence (25–81), days to maturity (50–140), number of grains/plant (9,000–90,000), plant height (20–156 cm), number of tillers/plant (5–35), panicle type (from very loose to very compact), flag leaf area (2–26 cm^2^), culm diameter (1.2–5 mm; Ketema, 1993; Assefa et al., 2001a,b). Variability in tef germplasm for culm internode diameter is a key factor in the identification of tef lines with improved lodging resistance.

Soon after the discovery of breeding techniques for tef (Berhe, 1975), several studies were made to investigate the inheritance of key agronomic traits and their contributions to tef breeding. The initial studies dealt with investigations of the inheritance of lemma color, seed color, panicle form in F_2_ and F_3_ populations of crosses involving genotypes with contrasting phenotypes (Berhe et al., 1989a,b,c). Subsequent studies were made by several other researchers (Tefera et al., 2003a,b, 2008; Chanyalew et al., 2006; Yu et al., 2006; Zeid et al., 2011).

### Diversity due to Agro-Ecology

Significant clinal diversity was reported in tef germplasm populations collected from different altitudinal zones for traits such as days to maturity, number of culm nodes, first and second basal culm internode diameter, and harvest index (Assefa et al., 2001b). Likewise, significant altitude-based diversity in tef germplasm populations was found for traits such as main shoot culm node number, days to maturity, diameters of the first and second lowest primary shoot culm internodes, and harvest index (Assefa et al., 2002a). However, no significant differences for qualitative traits (such as lemma, seed and anther colors and panicle form) were reported among the altitudinal zones (Kefyalew et al., 2000). On the other hand, for the trait days to maturity, 36 heterogeneous tef populations had lower diversity levels for accessions collected between altitudes of 1800 and 2400 m, while high diversity was noted for accessions obtained below 1800 m above sea level (Assefa et al., 2000).

Evaluations of 70 accessions of tef collected from different regions of Ethiopia showed significant variations within populations, among populations within regions, and among regions in most of the phenotypic traits (Tadesse, 1993). On the other hand, studies based on evaluations of 3600 tef lines representing 36 populations collected from the Central and Northern Regions of Ethiopia revealed significant regional diversity for seed color and days to maturity (Kefyalew et al., 2000). Furthermore, other studies showed significant regional diversity for lemma color, number of culm internodes, and counts of basal and middle spikelet florets in tef germplasm populations from different parts of the country (Assefa et al., 2002b).

An experiment at two locations using 144 accessions collected from different regions of Ethiopia showed that accessions from the same origin clustered into different classes and those from different origins also clustered into the same group (Adnew et al., 2005). Other studies further confirmed that the level of genetic diversity is higher in tef germplasm within a region than between regions, and as a result, accessions that had originated from the same region and altitude were grouped into distinct and distant clusters (Assefa et al., 2001b).

On the other hand, no significant differences were obtained among diverse altitude zones for parameters like days to panicle emergence, culm and panicle length, number of panicle branches, counts of fertile florets/spikelet, and shoot biomass (Assefa et al., 2001a,b). Moreover, diversity studies using 33 accessions collected from North-Western Ethiopia and four improved varieties (Ayalew et al., 2011) and selected tef genotypes (Plaza-Wüthrich et al., 2013) revealed considerable variations among the genotypes for many of the traits assessed.

However, this genetic variability is rapidly declining as farmers are quickly adopting improved cultivars and using them instead of landraces. In order to reduce the expected genetic erosion, the EIB has made rescue collections from different agro-ecological zones.

## Molecular Diversity in Tef

In the past, efforts have been made to characterize and analyze the diversity levels in cultivars of tef and its relatives based on approaches other than morphological or phenotypic data (**Table 3**). Before high-throughput sequencing provided copious amounts of molecular data, chromatography, flow cytometry, gel electrophoresis, and polymorphism assays were used for the molecular characterization of genetic diversity.

**Table 3:** Studies made on molecular (genotypic) diversity in tef.

Germplasm or genotypes		Technique	Reference
Type	Number		
Natural accessions	11	biochemical	Bekele and Lester (1981)
Natural accessions	37	Protein markers	Bekele et al. (1995)
Natural accessions	47	AFLP	Bai et al. (1999b)
RIL (Kaye Murri × Fesho)	85	AFLP	Bai et al. (1999a)
Natural accessions	47	RAPD	Bai et al. (2000)
Natural accessions	14	AFLP	Ayele et al. (1999)
Natural accessions	6	Diverse^∗^	Espelund et al. (2000)
RIL (Kaye Murri × *E. pilosa*)	116	RFLP	Zhang et al. (2001)
Natural accessions	92	ISSR	Assefa et al. (2003a)
RIL (tef ×*E. pilosa*)	124	AFLP, EST, ISSR, SSR	Chanyalew et al. (2007)
RIL (Kaye Murri × *E. pilosa*	94	Diverse markers^∗∗^	Yu et al. (2006)
Natural + improved	326	SSR markers	Zeid et al. (2012)
Natural accessions	31	Haplotype analysis and LD	Smith et al. (2012)
Natural accessions	20	SSR	Cannarozzi et al. (2014)

^∗^chloroplast DNA, 18S r, VP1 DNA.

^∗∗^AFLP, EST-SSR, SNP/INDEL, IFLP, ISSR.

### Proteins as a Marker

Early work using differences in protein content to classify and distinguish different accessions of tef employed the chromatography and electrophoresis of proteins involved in traits of interest such as seed storage proteins. Studies on the relatedness between *Eragrostis* species and tef accessions using chromatography of leaf phenolics and electrophoresis of seed proteins as biochemical markers showed complex patterns of variation amongst tef cultivars (Bekele and Lester, 1981). Similarly, polymorphisms among tef seed storage proteins (albumin, globulin, and prolamin) were found based on SDS-PAGE (Bekele et al., 1995). The study was able to classify 37 cultivars into seven groups, and suggested that the polymorphisms in albumins and globulins could be exploited to identify genotypes with desirable nutritional qualities.

### Genomics

**FIGURE 2:**
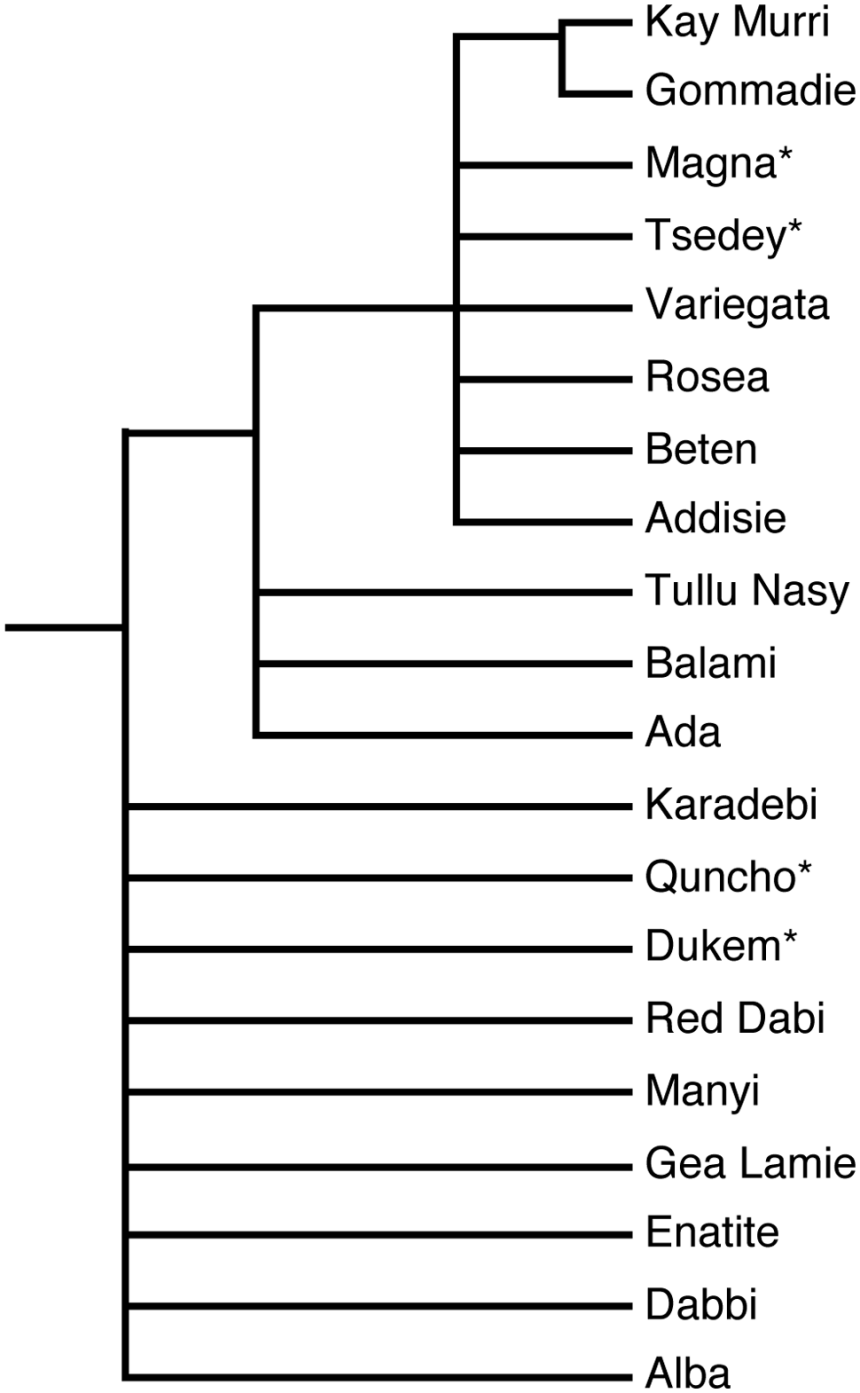
**Phylogenetic tree showing the relationship among natural accessions and improved varieties of tef.** The ^∗^ represents improved varieties. The phylogenetic tree was constructed from ∼200 bp surrounding an SSR marker located on linkage group nine (Zeid et al., 2011). Quncho, the most popular variety in Ethiopia was produced from a cross between the high-yielding Dukem variety and the white-seeded Magna variety.

Finding and exploiting DNA sequence variation within a genome is of utmost importance for crop genetics and breeding (Varshney et al., 2009). Over the last three decades, different methods have been developed to detect and quantify the genetic diversity of tef. The first techniques employed were flow cytometry, sequencing of single genes or regions and genotyping using AFLP, RAPD, RFLP, inter-simple sequence repeat (ISSR), and simple sequence repeat (SSR) markers, and these have all shed light on the structure of allelic diversity within selected tef germplasm collections (Girma et al., 2014). As shown in **Figure 2**, an SSR marker was used successfully to study the relationships among diverse tef genotypes, including natural accessions and improved varieties. However, only a small part of the diversity has been studied, and many of the essential questions still remain unanswered. Currently high-throughput single nucleotide polymorphism (SNP) genotyping is one of the methods that has been used to detect and exploit the genetic diversity of several crops. Genetic diversity analysis in some of the agriculturally important food crops such as sorghum (Nelson et al., 2011) and (Morris et al., 2013), barley (Close et al., 2009), rice (Thomson et al., 2012), bread wheat and emmer wheat (Akhunov et al., 2009), durum wheat (Trebbi et al., 2011), and maize (Yan et al., 2010) have been carried out with SNP genotyping methods employing next generation sequencing technologies.

#### Genome Size and Ploidy Determination

The genomic content of tef was first studied using flow cytometry, a popular method for ploidy screening and genome size estimation (Dolezel and Bartos, 2005). In the first measurement using four tef cultivars, the genome size was found to be between 714 and 733 Mbp (Ayele et al., 1996), relatively small for a grass (**Table 4**). The small genome size of tef made it a good candidate for genetic mapping and later genome sequencing. In addition, 32 of the first 35 tef ecotypes characterized (Ebba, 1975) as well as three commercial varieties were tested for ploidy level; all were tetraploid. In a another study with 10 released varieties of tef, following optimization of the flow cytometry conditions, the resulting genome size estimates were between 648 and 926 Mbp (Hundera et al., 2000; **Table 4**).

**Table 4:** Variations in 2C DNA content and genome size among tef genotypes.

Genotype	Seed color	Panicle form	2C DNA content (pg)	1C genome size (Mbp)	Reference
Burssa	White	Semi compact	1.48 ± 0.02	714	Ayele et al. (1996)
Fesho	Brown	Very loose	1.51 ± 0.03	729	Ayele et al. (1996)
Key Murri	White	Very compact	1.50 ± 0.04	724	Ayele et al. (1996)
Trotteriana	Brown	Very compact	1.52 ± 0.03	733	Ayele et al. (1996)
DZ-01-354	Pale white	Very loose	1.34 ± 0.67	648	Hundera et al. (2000)
DZ-01-974	White	Very loose	1.49 ± 0.75	719	Hundera et al. (2000)
DZ-01-787	White	Very loose	1.49 ± 0.75	719	Hundera et al. (2000)
DZ-01-99	Brown	Very loose	1.65 ± 0.83	798	Hundera et al. (2000)
DZ-01-196	Very white	Fairly loose	1.68 ± 0.84	811	Hundera et al. (2000)
DZ-Cr-37	White	Very loose	1.61 ± 0.80	772	Hundera et al. (2000)
DZ-Cr-82	White	Very loose	1.92 ± 0.96	926	Hundera et al. (2000)
DZ-Cr-44	White	Very loose	1.78 ± 0.89	859	Hundera et al. (2000)
DZ-Cr-255	White	Very loose	1.52 ± 0.77	754	Hundera et al. (2000)
DZ-Cr-358	White	Very loose	1.38 ± 0.69	666	Hundera et al. (2000)

#### Sequence-Based Diversity

Around the same time, sequencing of single genes and small genomic regions was also employed to measure diversity and genetic relationships. Sequence analysis of non-coding regions of chloroplast DNA, 18S rDNA, and the transcription factor VP1 did not show significant intra-specific variation among six tef cultivars (Espelund et al., 2000). In addition, two rht1 (reduced height) gene homologs and three sd1 (semi-dwarf) genes were later sequenced for 31 accessions of tef (Smith et al., 2012). A low level of nucleotide diversity was observed and the genetic diversity could be organized into 2–4 haplotypes, a relatively small number.

#### Molecular Markers

Molecular markers are short sections of DNA that differ between varieties, and thus can be used for identification of a germplasm by a specific pattern of polymorphisms, to assess diversity and to determine relationships. Genetic relationships among accessions of *E. tef*, *E. pilosa,* and *E. curvula* which were collected from Ethiopia and USA were assessed based on AFLP (Bai et al., 1999b; Ayele and Nguyen, 2000) and RAPD markers (Bai et al., 2000). These analyses depicted relatively low levels (18%) of polymorphism within *E. tef*, and high similarity between *E. tef* and *E. pilosa*. The Jaccard similarity coefficient (size of the intersection of two sets divided by the size of the union) among two tef populations ranged from 84 to 96% for RAPD and from 73 to 99% for AFLP markers, indicating very close similarity among accessions. On the other hand, ISSRs analysis on 92 tef genotypes from seven regions plus improved varieties showed higher diversity among tef cultivars with Jaccard similarity coefficients ranging from 26 to 86% (Assefa et al., 2003a). A comparison of AFLP, EST-SSR, ISSR, and SSR markers for polymorphisms in tef recombinant inbred lines concluded that EST-SSR and ISSR makers had as much polymorphism as AFLP markers (Chanyalew et al., 2007).

Assessment of genetic diversity and relationships among 326 tef accessions, 13 wild relatives, and four commercial varieties from the United States based on 39 SSR markers, 26 of which were flanking QTL intervals for stem strength related traits, yield and lodging index showed genetic similarity (GS) estimates of between 0.20 and 0.99 among tef accessions (Zeid et al., 2012), and this contrasted with the narrow genetic background suggested in the earlier studies described above. A large base of genetic diversity is indispensable for successful breeding programs. However, the diversity in tef has never been sufficient to produce the desired improvement in lodging resistance. Given the complexity of lodging and its component traits such as plant height, and culm internode length and diameter, alternative approaches including genetic transformation in line with marker-assisted selection should be considered for improving the malignant lodging syndrome in tef.

The afore-mentioned study of Zeid et al. (2012) also revealed 27 cases where accessions were identical to one or more of the other accessions. According to the authors, the high GS estimates from previous studies (Ayele et al., 1999; Bai et al., 1999a, 2000; Ayele and Nguyen, 2000) using the same plant material (landraces), was a marker dependent issue rather than due to low polymorphism in tef as previously suggested. An SSR marker used to construct a phylogenetic tree for 16 natural accessions and four improved varieties of tef showed the relationship among these genotypes (Cannarozzi et al., 2014). A multiple sequence alignment of approximately 200 base pairs was variable at 32 sites of which 25 were informative for determining evolutionary relationships.

#### Genetic Mapping

Genetic maps show the position of the molecular markers and QTLs relative to each other in terms of recombination frequency, and are used to find genes responsible for traits of interest. The first genetic map of tef was produced with an intra-specific cross between the ‘Kaye Murri’ and ‘Fesho’ cultivars and contained 211 AFLP markers in 25 linkage groups (Bai et al., 1999a). The low number of polymorphisms found between the two varieties of tef impeded its use in breeding. The same group later produced an RFLP linkage map using 116 RILs from the cross of ‘Kaye Murri’ with *E. pilosa* (Zhang et al., 2001). This inter-specific cross produced far more polymorphisms; however, the level of polymorphism was still smaller than that of other grasses.

The group of Sorrells has been instrumental in identifying QTLs associated with yield related traits and producing genetic maps of tef using RILs from a cross between ‘Kaye Murri’ and *E. pilosa* with a variety of markers (Chanyalew et al., 2005; Yu et al., 2006; Zeid et al., 2011). Clusters of QTLs controlling yield and plant architecture were identified, thereby forming useful targets for applied breeding.

#### High-Throughput Genomics

During the last 5 years, tef genomics research has moved from analysis of a handful of genetic polymorphisms, toward whole genome sequencing and genome-wide polymorphism search. The genome and the transcriptome of the tef genotype Tsedey (DZ-Cr-37) were sequenced by the Tef Improvement Project at the Institute of Plant Sciences, University of Bern (Cannarozzi et al., 2014). Genome sequencing has many applications in tef improvement. First and foremost, primer sequences can be identified without resorting to other genomes or degenerate primers. This is especially important for the isolation of homeologous copies of each sub-genome for techniques such as Targeting Induced Local Lesions IN Genome (TILLING) which require genome specific primers. The genome has already been used to discover genetic markers such as SNPs and SSRs useful for marker-assisted breeding, for the construction of high density genetic maps and for linkage disequilibrium studies on diverse germplasm. Possession of the genomic sequences allows an understanding of the molecular basis of the mechanisms of tef’s many desirable properties such as its tolerance to many abiotic and biotic stresses. The genes obtained from these analyses could be then transferred to other economically important crops.

### Transcriptomics

To date, the transcriptome from only one tef improved variety has been sequenced (Cannarozzi et al., 2014), precluding comparison of transcriptomes between varieties or accessions. For the Tsedey improved variety (DZ-Cr-37), a normalized transcriptome library was prepared and sequenced from roots and shoots of tef seedlings resulting in a transcriptome with 27756 gene clusters and 38333 transcripts. In addition, a second non-normalized library was obtained from various tef tissues subjected to drought and water-logging, resulting in a similar number of gene clusters.

An RNA-Seq study of two different varieties of quinoa (Raney et al., 2014), one representing valley ecotypes and another one representing high plains ecotypes, under different watering conditions was recently conducted. It was found that 27 putative gene products were differentially expressed based on variety × treatment interaction. These included significant differences in root tissue in response to increasing water stress. A similar strategy could be employed for tef varieties to discover the QTLs responsible for specific accessions’ traits.

### Proteomics

Proteomics has emerged as an indispensable tool to analyze the whole or specific protein complement present in a particular tissue, organ, cell, or organelle (Agrawal et al., 2005; Benkeblia, 2011). In recent years, plant proteome analysis has evolved into high-throughput techniques resulting in the generation of high quality data with the continuous improvements made in sample preparation, protein separation, mass spectrometry, and protein search algorithms (Thelen, 2007; Benkeblia, 2011).

The application of proteomic studies has led to the discovery of a number of important proteins, and has facilitated attempts to explore their importance in improving plant yield and tolerance to environmental stresses (Salekdeh and Komatsu, 2007; Mochida and Shinozaki, 2010; Benkeblia, 2011). Similarly, to take advantage of the diversity among tef lines, proteomic approaches can be narrowed and refined to investigate which proteins are characteristic of specific lines or play important roles in a selected tef line. The corresponding genes of these proteins of interest can then be isolated and characterized from the tef genome provided it is comprehensively annotated. The particular phenotype conferred by the protein(s) of interest can then be introduced or enhanced in other tef lines using genetic and transgenic approaches to improve crop productivity. This functional genomics approach has been proposed as a standard ‘omic’ strategy for the improvement of many crop species (Agrawal and Rakwal, 2006).

To date, there has been no published proteomic study on tef with respect to protein profiling or comparative proteomics, while numerous such studies have been done on maize (Mohammed, 2005; Zhu et al., 2006; Prinsi et al., 2009), wheat (Jiang et al., 2012; Budak et al., 2013) and rice (Agrawal and Rakwal, 2006; Kim et al., 2014) using both gel-based and gel-free (mass spectrometry) techniques. Recently, proteomic profiling of the tef drought response has been undertaken, and should contribute valuable information on the key biological processes affected by water loss in tef (Kamies et al., 2014).

A key constraint affecting tef yield is salinity in the lowland and Rift Valley areas of Ethiopia, especially in the Awash valley and lower plains (Asfaw and Dano, 2011). The effects of increased salinity on tef yield and yield components were investigated by screening 15 lowland tef genotypes (10 accessions and 5 varieties) at different salinity levels. They found grain yield per main panicle to be the most affected by increased salinity, and although there were differences in genetic variation between tef varieties and accessions, salt tolerance was observed in accession 237186 and variety DZ-Cr-37 (Tsedey) genotypes (Asfaw and Dano, 2011). This particular variety of tef, thus requires further proteomic and metabolomic investigation in order to elucidate the mechanisms of salt tolerance in tef and for identification of salt tolerant markers.

A comparative proteomics approach could be employed to investigate the cell wall proteome in both the tef stem and root tissues. A similar comparative proteomic study was done on maize primary and lateral roots whereby proteins involved in cell wall metabolism, cell elongation, lignin metabolism, defense, and citrate cycle were identified (Liu et al., 2006; Zhu et al., 2006). Such a study can be done on tef to identify and characterize stress-related cell wall proteins.

It is important to note that future tef improvements using the ‘omics’ tools should be conducted on one standardized consensus tef variety to allow for ease of comparison across functional genomic studies and to facilitate interpretation of data. Many studies have been conducted on the improved variety DZ-Cr-37 mostly because it is grown in areas which receive low rainfall (especially terminal drought-prone areas), and has been proposed to have a degree of drought tolerance in addition to being widely adaptable to differing climates (Ayele et al., 2001; Assefa et al., 2003a, 2011; Admas and Belay, 2011; Cannarozzi et al., 2014). Furthermore, since the genome and transcriptome information of this variety is available (Cannarozzi et al., 2014), it provides a platform for different proteomic strategies such as sub-cellular proteomics or phospho-proteomics to investigate stresses associated with tef. As stated earlier, proteomics is a functional tool that can provide insight to phenotypes of interest, and is largely dependent on the level of clarity and surety provided by the databases generated and the level of annotations made to the sequences. Since tef genome sequencing has been conducted and database annotation is in its infancy, proteo-bioinformatic approaches are somewhat limited, which in time will be remedied as more and more protein sequences are curated.

## Tef Diversity in Key Traits

### Grain Yield and Shoot Biomass

Development of varieties with high grain yield has been one of the top priorities of the National Tef Improvement Program in Ethiopia (Assefa et al., 2011). This varietal development process depends on the variability available within the gene pool. Over the past three decades, several studies (Assefa et al., 1999, 2000, 2001b, 2003b; Teklu and Tefera, 2005) were conducted to assess this variability, and tests both at research stations and on-farm yield trials were carried out at various locations. Over 30 improved varieties have been developed pushing the national average tef yield from 0.7 t/ha in 1994 to 1.5 t/ha in 2013 (CSA, 2014) hinting that the yield potential in tef can be further exploited. Variability in shoot biomass has also been studied in the majority of the above-mentioned studies, and a wide range (4–105 g/plant) was reported, suggesting the presence of high variability for this trait within the tef gene pool.

### Seed Size and Seed Coat Characteristics

Despite the importance of seed size in terms of both agronomy and productivity, there exists only one study on the variability of seed size in tef. Using two improved tef genotypes, sieve-graded larger tef seeds had an increased seed yield, but it was concluded that this increase did not justify seed grading in tef (Belay et al., 2009). Seed coat characteristics in tef have received little research attention. The only study reported in literature showed slime cell differences in two tef genotypes and a wild *Eragrostis* species (Kreitschitz et al., 2009). The authors reported the presence of slime cells, a type of modified epidermal cells covering the fruit of the genotypes under investigation, and that such cells could play an adaptive role for tef plants growing in dry areas.

### Physiology and Agronomy Related Traits

Due to a growing interest in utilizing tef as a gluten-free alternative to rice, there is corresponding interest in producing tef at a larger scale in some western countries. However, as a short day tropical cereal, growing tef in the temperate regions during the summer when the days get longer poses a big challenge. In order to investigate the ability of tef to flower in response to changes in the photoperiod, the effect of the relative lengths of day and night using four tef cultivars were studied. Two of the four cultivars had a stronger photoperiod response; panicle initiation as well as development and outgrowth of the panicle were influenced by photoperiod (van Delden et al., 2012).

#### Nitrogen-Use Efficiency

Nitrogen use efficiency (NUE), defined as the ratio of grain yield to supplied N, is a key parameter for evaluating a crop cultivar, and it is composed of N uptake efficiency and N physiological use efficiency (de Macalel and Vlek, 2004). Breeding for NUE in tef could play a considerable role in reducing the amount of nitrogen fertilizer applied without affecting yield significantly. The NUE of tef is very low, ranging from 16 to 34% (Tulema et al., 2005). In the last decade, some authors looked at the genetic variation in NUE of tef (Tulema et al., 2005; Balcha et al., 2006; Habtegebrial et al., 2007). We suggest that further comparisons of nitrogen-use efficiency within the tef gene pool are important to evaluate their performance under limited nitrogen supply.

#### Osmotic Adjustment and Root Depth

Water deficit and salinity are among the abiotic production constrains limiting survival, growth, and productivity of tef. However, it is likely that there exists variability within the tef germplasm pool, and certain tef genotypes could adopt some strategies such as osmotic adjustment to resist these constraints. Systematic sampling of 54 tef genotypes from the entire gene pool showed a significant genotype effect on osmotic adjustment and root depth, irrespective of the area from where the genotypes were collected (Ayele et al., 2001).

### Stress Related Traits

#### Drought Tolerance

The production areas of tef range from the cool highlands to the dry lowlands that are often associated with moisture deficit during critical stages of plant development. Studies investigating the effect of moisture deficit on the performance of tef plants range from variability in key characters and response studies (Degu et al., 2008; Mengistu, 2009; Ginbot and Farrant, 2011; Shiferaw et al., 2012) to mapping QTLs related to economically important traits under water deficit conditions (Degu, 2010). In general, the majority of the studies have shown that there is genetic variability among the genotypes investigated suggesting that the tef gene pool harbors moisture stress tolerant genotypes that could be screened through efficient tools such as molecular markers.

#### Salinity and Acidity Tolerance

Due to the anticipated changes in the climate and expansion of farmlands in the rift valley areas, studying and documenting the effect of such growing conditions on tef production and productivity is worthwhile. Earlier, a few of such studies have been published including one which showed the presence of broad intra-specific variability among the ten tef accessions studied for salinity tolerance (Asfaw and Dano, 2011), and one which showed the presence of genetic variability for tolerance to soil acidity and aluminum toxicity in selected tef genotypes (Abate et al., 2013).

### Nutrition, Health, and Consumers’ Preference Related Traits

#### Seed Color Consumers’ Preference

The Ethiopian Standards Agency recognizes four classes of tef grain mainly based on color of the seed (QSAE, 2001). These are very white, white, brown and mixed (commonly known as *Sergegna*). Oftentimes, farmers produce brown-seeded types for home consumption and white types for sale. Assessment of the diversity patterns of the seed color in tef with respect to growing regions and altitude zones revealed that the majority of tef collections from the north and northwestern part of Ethiopia were white-seeded as compared to those from the southern part of the country which were brown-seeded (Assefa et al., 2002b).

#### Nutritional Quality and Physico-Chemical Properties of Tef Seed

Knowledge of the physical properties of tef seed can be useful for agronomy, storage, marketing, and other socio-cultural purposes. A handful of studies have been carried out on the starch and protein contents of tef seed. Starch is the principal carbohydrate of all cereals, and represents, from 56% (oats) to 80% (maize) of the grain dry matter (Eliasson and Larssson, 1993). The starch characteristics of tef seed have been extensively studied (Bultosa et al., 2002, 2008; Bultosa and Taylor, 2003, 2004; Bultosa, 2007). The scientific study of tef grain protein and more specifically the amino acid composition extends back for over 50 years. Previous, reports indicated that tef seed contains a good balance of the essential amino acids, except lysine (Jansen et al., 1962). Three decades later, investigations of the polymorphism of seed albumin, globulin, and prolamin fractions showed the existence of considerable polymorphism in the studied protein fractions among the 37 tef cultivars investigated (Bekele et al., 1995). At the same time, Tatham et al. (1996) purified and characterized prolamins of tef. According to this study, the tef protein is made up of 9–14% prolamins and these are similar to prolamins of maize and sorghum. This value is in a similar range to the previous results (3–15%; Bekele et al., 1995). However, according to a recent report, the prolamin content of three tef genotypes studied reached as much as 40% (Adebowale et al., 2011). In these studies, there is a discrepancy between the number of genotypes used and the methods employed. Clearly variability exists within the tef gene pool and a comprehensive study with more genotypes and modern tools to characterize and document the seed protein fractions is necessary. More recently, studies on tef seeds have changed course and three studies by Gebremariam et al. (2013a,b,c) investigated the malt quality attributes, while another by Boka et al. (2013) assessed the antioxidant properties of differentially processed tef grain.

As a potential alternative gluten-free food source for celiac patients, tef has been studied along with wheat, oat rye, barley, rice, maize, and triticale (Spaenij-Dekking et al., 2005). This study showed that the tef cultivars evaluated contained no gluten or gluten homologs. This is the first scientific evidence for the absence of gluten in tef flour. Recently, this has been supported by results from the genome sequence initiative (Cannarozzi et al., 2014).

## Conclusion

The broad spectrum of trait diversity in tef implies great opportunities for genetic improvement through either direct selection or intra-specific hybridization between parental lines with desirable traits. In addition, statistical tools such as correlation analysis can be used to aid selection of candidates in breeding programs. Additionally several mutagenized populations have been developed to supplement the natural diversity present in tef. As some studies reviewed here, used only few or selected tef genotypes, they may not be representative of the existing diversity in tef accessions. Future research is required to explore diversity in different traits of agronomic and nutritional importance. Concerted efforts of all stakeholders in research, development and funding are required to promote the research and development of vital crops such as tef in order to promote food and nutrition security.

## Conflict of Interest Statement

The authors declare that the research was conducted in the absence of any commercial or financial relationships that could be construed as a potential conflict of interest.
